# Telitacicept in Recurrent IgA Nephropathy After Kidney Transplantation: Encouraging Signal, Cautious Interpretation

**DOI:** 10.1016/j.ekir.2026.106554

**Published:** 2026-04-20

**Authors:** Bobby Chacko

**Affiliations:** 1 Nephrology and Transplantation Unit, John Hunter Hospital, Newcastle, New South Wales, Australia; 2 School of Medicine and Public Health, University of Newcastle, New South Wales, Australia

See Clinical Research on Article 106527

Recurrent IgA nephropathy (rIgAN) after kidney transplantation continues to pose a major challenge in long-term allograft care, even though overall transplant outcomes have steadily improved. IgA nephropathy (IgAN) remains one of the most frequently recurring primary glomerular diseases in the posttransplant setting, with reported recurrence rates between 20% and 60%. Among those who develop clinically meaningful recurrence, as many as 30% eventually progress to graft failure.^1^ These figures underscore a central reality: transplantation replaces the kidney; however, it does not correct the systemic immune dysregulation that drives IgAN in the first place.

The persistence of rIgAN reflects its intrinsic pathophysiology rather than alloimmune injury. The well-established multihit model of IgAN remains fully operative after transplantation. Patients continue to produce excess galactose-deficient IgA1, generate antiglycan autoantibodies, form circulating immune complexes, and deposit these complexes within the mesangium, triggering inflammatory and complement-mediated injury.^2^ None of these processes are reset by transplantation. The newly transplanted kidney is exposed to the same pathogenic milieu that damaged the native kidneys. Because conventional maintenance immunosuppression is designed primarily to prevent rejection rather than to modulate mucosal immune pathways, it exerts limited influence on the mechanisms that drive recurrence. The relative immunologic protection of the mucosal compartment further explains why standard transplant immunosuppression fails to prevent disease reemergence.^3^

Clinically, rIgAN presents with considerable heterogeneity. Some cases are detected incidentally on protocol biopsies showing mesangial IgA deposition without overt clinical manifestations. Others present with proteinuria, microscopic hematuria, or a slow but progressive decline in estimated glomerular filtration rate.^2^ A minority of patients develop rapidly progressive crescentic disease. Among these features, proteinuria remains the most widely used surrogate marker of disease activity and a strong predictor of long-term graft outcomes. Its trajectory often guides both clinical decision-making and assessment of therapeutic response.

Management of rIgAN remains largely supportive and is heavily informed by experience in native kidney disease. Renin-angiotensin system blockade continues to serve as the foundation of therapy, complemented by strict blood pressure control and dietary sodium restriction. The role of sodium-glucose cotransporter-2 inhibitors in transplant recipients is evolving; though these agents have demonstrated benefit in native IgAN, their use in the transplant population requires individualized assessment because of concerns about infection risk and hemodynamic effects. In addition, maintenance immunosuppression influences recurrence risk. Observational studies consistently suggest that early corticosteroid withdrawal is associated with higher recurrence rates and worse graft outcomes, supporting the continued use of low-dose corticosteroids when clinically appropriate.^4^

In recent years, the therapeutic landscape has begun to shift toward targeted interventions that address disease-specific mechanisms. Much of this momentum comes from clinical trials in native IgAN, with emerging but still limited data in transplant recipients. Gut-directed targeted-release budesonide, which reduces galactose-deficient IgA1 production by modulating mucosal immunity, has demonstrated proteinuria reduction in native disease and early transplant case series.^5^ Complement-directed therapies are also gaining traction. Iptacopan, a factor B inhibitor, has shown efficacy in high-risk native IgAN and early promise in transplant recipients, although treatment response may vary depending on complement phenotype.^6^ Increased deposition of complement components such as C3, C1q, and C4d is well-documented in rIgAN, and C4d positivity in particular has been associated with a higher risk of graft loss. These observations reinforce the relevance of complement-mediated injury in recurrent disease.

Within this evolving therapeutic framework, B-cell–directed therapies represent a biologically rational strategy because of the central role of abnormal IgA production. Rituximab, widely used in transplantation for other indications, has been explored in IgAN. In a randomized study of 64 transplant recipients with biopsy-proven rIgAN, rituximab therapy led to complete remission in 38% of treated patients compared with none in the control group, and a greater proportion achieved reductions in proteinuria at 12 months.^7^ Biopsies from rituximab-treated patients showed less endocapillary hypercellularity despite persistent IgA deposition.^7^ However, in native IgAN, rituximab has generally shown limited efficacy, likely because of the prominent role of mucosal lymphoid tissue—particularly intestinal lymphoid aggregates—which may be relatively resistant to systemic B-cell depletion.

This has led to growing interest in therapies targeting the BAFF and APRIL pathways, which are critical for B-cell maturation, survival, and plasma-cell differentiation. Telitacicept, a recombinant fusion protein that inhibits both BAFF and APRIL, offers a more direct approach to reducing pathogenic IgA production. Early retrospective data in transplant recipients have been encouraging. In a small cohort of 10 patients with rIgAN, telitacicept was associated with reductions in proteinuria and an acceptable safety profile.^8^

The prospective single-center cohort study by Sui *et al.*^9^ adds important prospective evidence to this emerging field. The study enrolled 25 kidney transplant recipients with biopsy-confirmed rIgAN and administered telitacicept as add-on therapy: 240 mg weekly for 12 weeks, followed by dosing every 2 weeks until week 28. The primary end points were changes in 24-hour urinary protein and urine albumin-to-creatinine ratio, with secondary end points, including remission rates, kidney function, laboratory parameters, and mechanistic biomarkers.

Of the 25 enrolled patients, 22 completed the 28-week follow-up. By week 28, telitacicept therapy was associated with a 51.6% reduction in 24-hour urinary protein and a 62.7% reduction in urine albumin-to-creatinine ratio, both statistically significant. These reductions are clinically meaningful given the strong association between proteinuria and graft outcomes. Complete remission occurred in nearly half of the patients, and partial remission in more than half, indicating that a substantial proportion experienced measurable improvement. Importantly, kidney function remained stable throughout the study, with no significant decline in estimated glomerular filtration rate.

Mechanistic analyses further strengthened the biological plausibility of the observed clinical effects. Circulating levels of galactose-deficient IgA1 and asialoglycoprotein receptor decreased following treatment, consistent with inhibition of BAFF or APRIL signaling and downstream modulation of IgA production. These biomarker changes suggest that telitacicept is exerting on-target immunologic effects and provide a mechanistic link between therapy and disease-specific pathways.

The short-term safety profile appeared acceptable, with no treatment-related serious adverse events observed. Telitacicept was generally well-tolerated; however, the small cohort size and limited duration of follow-up make it difficult to fully evaluate longer-term risks such as infection, hypogammaglobulinemia, or malignancy; particularly given the additive immunosuppression layered onto standard transplant therapy. As with any immunomodulatory agent, extended monitoring will be essential to define its true safety profile over time.

Despite these encouraging findings, several limitations warrant caution. The single-arm design without a control group limits causal inference. rIgAN is characterized by variability in disease activity, and reductions in proteinuria may reflect regression to the mean or delayed effects of prior therapy. Although the study ensured stability of background treatment, residual confounding cannot be excluded. Moreover, the reliance on short-term surrogate end points limits the ability to assess long-term outcomes such as sustained estimated glomerular filtration rate preservation, histologic progression, or graft survival. The durability of response beyond 28 weeks remains unknown.

For these reasons, the findings should be viewed as hypothesis-generating rather than practice-changing. The study provides promising early evidence that telitacicept may reduce proteinuria, stabilize graft function, and modulate disease-related biomarkers in rIgAN. However, confirmation in larger, multicenter randomized controlled trials with longer follow-up is essential before telitacicept can be integrated into routine clinical practice.

Several key uncertainties remain. Determining which patients with rIgAN are most likely to benefit, clarifying the optimal timing and duration of treatment, and defining how telitacicept compares with; or potentially complements; other emerging therapies are all essential next steps. The prospect of biomarker-guided treatment is particularly compelling, though it requires more rigorous validation before it can be integrated into clinical practice.

In summary, rIgAN remains a significant and therapeutically challenging condition in kidney transplant recipients. Current management is largely supportive and does not adequately address the underlying disease mechanisms. Telitacicept represents a promising targeted approach supported by mechanistic rationale and early clinical data. The prospective study by Sui *et al.*^9^ adds valuable evidence but must be interpreted with caution. Until more robust data are available, management should remain individualized, guided by clinical and histologic parameters, with strong consideration for clinical trial enrollment.

## Disclosure

The author declared no competing interests.
